# Effects of mandarin peel powder on growth, biochemical, immune, and intestinal health in *Oreochromis niloticus* at suboptimal temperatures

**DOI:** 10.1186/s12917-024-04273-8

**Published:** 2024-10-02

**Authors:** Rasha M. Reda, Mostafa I. Abd El-Rahim, Dawlat A. Elkerdawy, Mohamed M. M. Metwally, Nermin Said

**Affiliations:** 1 Department of Aquatic Animal Medicine, Faculty of Veterinary Medicine, Zagazig University, PO Box 44511, Zagazig, Sharkia Egypt; 2 Department of Animal and Poultry Production, Faculty of Technology and Development, Zagazig University, PO Box 44511, Zagazig, Sharkia Egypt; 3 Department of Pathology and Clinical Pathology, Faculty of Veterinary Medicine, King Salman International University, Ras Sidr, Egypt; 4 Department of Pathology, Faculty of Veterinary Medicine, Zagazig University, PO Box 44511, Zagazig, Sharkia Egypt

**Keywords:** Growth performance, Mandarin peel, *Oreochromis niloticus*, Suboptimal temperature, Stress

## Abstract

This 60-day study aimed to examine the efficacy of a diet supplemented with mandarin peel powder (MP) in enhancing the health and survival of *Oreochromis niloticus* under suboptimal temperature conditions (21 ℃). One hundred and eighty Nile tilapia fish (22.51 ± 0.04 g) were randomly distributed into four experimental groups; each of 3 replicates (15 fish per replicate). The first group (CONT) received a basal diet without MP. The second (MP10%), third (MP15%), and fourth (MP20%) groups were fed diets containing 10, 15, and 20% MP powder, respectively. At the end of the feeding trail, growth performance, serum growth hormone, α-amylase enzyme, lysozyme activity, nitric oxide, protease activity, globulin, serum levels of IL-1ß, antioxidant status, and intestinal histology were measured. The results showed insignificant differences between CONT, MP15%, and MP20% groups in the final body weight and specific growth rate. The growth hormones in the MP15% and MP20% groups did not show a significant difference compared to fish fed a normal basal diet (CONT). However, the amylase enzymes were significantly greater in both groups. The MP20% and MP15% groups showed a significant increase in antioxidant, lysozyme, nitric oxide, and protease activities compared to CONT. The results also showed that fish that were fed a diet with MP had significantly less of the pro-inflammatory cytokine interleukin-1 beta, and their intestinal villi got wider, especially in the MP20% group. It could be concluded that feeding tilapia on a diet with 20% MP is an effective strategy to improve their health when the temperature is below 21 °C. This is because the fish exhibit higher levels of antioxidant activity, reduced pro-inflammatory responses, and improved intestinal health without difference in the growth performance in compared to control group.

## Introduction

Aquaculture is critical for global food security because it contributes significantly to global protein consumption and provides a consistent and often more sustainable supply of protein than wild fisheries [1–3]. Thus, aquaculture is regarded as one of the critical pillars that will achieve the Sustainable Development Goals (SDGs) by promoting food security and achieving SDGs (zero hunger) [4–6]. Nile tilapia holds great importance in this industry because of its fast growth, adaptability, and economic value [7, 8]. Nevertheless, the tilapia sector encounters difficulties associated with improper rearing circumstances and stress management, which can have a negative impact on the health and growth of tilapia [9–11].

One of the main issues in aquaculture is maintaining ideal temperature conditions, which are essential for fish physiological function and growth. Tilapia may experience stress, weakened immunological responses, and slower growth rates as a result of suboptimal temperatures [9, 12, 13]. Temperature control has historically mostly relied on energy-intensive techniques, which may not always be practical or sustainable [14–16]. Consequently, it is imperative to investigate novel strategies for overcoming this obstacle.

Agro-industrial wastes produce massive quantities of expensive and environmentally harmful fruit waste byproducts [17, 18]. Numerous studies have established that these wastes have a high nutritional value as feed additives for fish and livestock, which could enhance growth and immune status as well as serve as a replacement for some conventional feeds [19]. Fruit wastes can be used as cost-effective, innovative feed ingredients in fish and livestock rations, providing a sustainable solution to feed shortages that aligns with the United Nations Sustainable Development Goals (SDGs) [18, 20, 21]. This approach not only avoids food waste and promotes circular economy concepts, but it also lowers animal production costs, hence improving food security and economic resilience in rural areas [22].

Citrus wastes—orange, mandarin or tangerine, lime, lemon, and grapefruit—have a high biological value and may be beneficial to health [23, 24]. This is because they contain high-quality fiber, pectin, and bioactive substances like carotenoids and polyphenols, which are used in the food, pharmaceutical, and cosmetics industries [24–26]. Mandarin peel (MP) contains a variety of bioactive substances, including flavonoids, antioxidants, and essential oils. These chemicals have been proven to have antibacterial, anti-inflammatory, and immune-enhancing effects [27–29]. Citrus waste has been incorporated into aquatic feed in a variety of forms in prior studies, including peels, seeds, extracts, essential oils, and secondary plant metabolites [30–37]. These studies revealed that citrus compounds can enhance antioxidant activity, improve growth, and strengthen the immune system of various fish species such as Nile tilapia (*Oreochromis niloticus*), African catfish (*Clarias gariepinus*) [30], common Carp (*Cyprinus carpio*) [32, 38], rainbow trout (*Oncorhynchus mykiss*) [33, 34], gilthead seabream (*Sparus aurata*) [35, 39], rohu (*Labeo rohita*) [36], and silver catfish (*Rhamdia quelen*) [37].

The objective of this study was to examine the effectiveness of mandarin peel powder as a functional dietary supplement on the growth performance, growth hormone levels, amylase enzyme activity, antioxidant-immune response in *Oreochromis niloticus* under suboptimal temperature conditions.

## Materials and methods

### Collection and preparation of mandarin peel

In this study, mandarin peel (MP) was used, and it was acquired from a traditional market in Zagazig, Egypt. The peel underwent a thorough water wash before being allowed to dry for 15 days at 28 ± 2 °C without exposure to the sun. To make a very fine powder, the dried peel was ground in a pestle and mortar. The milled powder sample was divided into two parts, one of which was used as powder (MP) and kept in a sealed vial at 4 °C. The other portion of the milled powder sample was used for extraction following a method described by Ho and Kuo [40] to identify the bioactive components in MP.

### Determination of bioactive compounds in mandarin peel extract (MPE) using gas chromatography-mass spectrometry (GC-MS)

The bioactive compounds in the MP extract (MPE) were identified via a Trace 1300 GC-TSQ mass spectrometer (Thermo Scientific, Austin, TX, USA) with a direct capillary column TG-5MS (30 m x 0.25 mm x 0.25 μm film thickness). The components were identified by comparison of their mass spectra with those of the WILEY and NIST mass spectral databases [41].

Table 1 displays the GC chromatogram of the mandarin peel (*Citrus reticulata* extract). Mandarin peel extract constituents were identified by comparing their spectrum data to those listed in the NIST 11(National Institute of Standards and Technology) Mass Spectral Database. As per Table 1, the predominant constituent of mandarin peel extract (*Citrus reticulata*) was found to be (Z., Z)-a-Farnesene (Rt = 13 min, peak area of 23.68%). This was followed by Benzoic acid, 2-(1-oxopropyl) (Rt = 19.62 min, peak area of 15.78%), and Bicyclo (7.2.0) undec-4-ene,4,11,11-trimethyl-8-methylene-, (1R- (1r*,4E,9s*) (Rt = 11.06 min, peak area of 13.59%). Peak areas for the remaining components were less than 10%.

**Table 1:** Composition of experimental diets (%)

Ingredients	Control (D1)	Diet 2 (D2)	Diet 3 (D3)	Diet 4 (D4)
Fish meal (65.4% CP)	23	23	23	23
Soybean meal (44% CP)	32	32	32	32
Yellow corn	24	24	19	18
Wheat flour	5	0	0	0
Mandarin peel	0	10	15	20
Wheat Bran	9	4	4	0
Fish oil	4.5	4.5	4.5	4.5
Methionine	0.5	0.5	0.5	0.5
Vitamin and mineral premix*	1	1	1	1
Dicalcium phosphate	1	1	1	1
**Chemical composition (air dry matter basis%)**				
Crude protein	33.20	31.10	32.80	31.40
Crude fiber	2.52	2.76	3.50	2.57
Ether extract	9.06	11.95	12.13	11.26
Carbohydrate	38.88	38.43	35.05	38.46
Moisture	9.78	9.31	9.85	9.69
Ash	6.56	6.45	6.67	6.62

*Vitamin mix (IU or mg kg diet): vitamin A, 16000 IU; vitamin D, 8000 IU; vitamin K, 14.72; thiamin, 17.8; riboflavin, 48; pyridoxine, 29.52; cynocobalamine, 0.24, tocopherols acetate, 160; ascorbic acid (35%), 800; niacinamide, 79.2; calcium-D- pantothenate,73.6; folic acid, 6.4; biotin, 0.64 L-carnitine, 100

*Mineral mix (mg kg diet): Cu (CuSO4), 2.0; Zn (ZnSO4), 34.4; Mn (MnSO4), 6.2; Fe (FeSO4), 21.1; I (Ca (IO3)2), 1.63; Se (Na2SeO3), 0.18; Co (CoCl2), 0.24; Mg (MgSO4.H2O), 52.7

### Experimental diets

According to the National Research Council [42], four experimental diets were formulated at the Fish Research Unit, Faculty of Veterinary Medicine, Zagazig University, Egypt (Table 2) that satisfied the tilapia fish’s dietary requirements. The first diet (D1) served as the baseline control diet without any supplements. Supplements of MP at 10, 15, and 20% were added to the second (D2), third (D3), and fourth (D4) diets, respectively. Each feed ingredient in the diet was combined, crushed, and formed into 1.5-mm-diameter pellets before being dried for a day at room temperature. Up until use, the pellets were kept at 4 °C.

**Table 2:** GC–MS chromatogram of mandarin peel (*Citrus reticulata*) retention time (min) and area (%) of the various compounds assigned in the extract

Retention Time (RT, min.)	Compound Name	Molecular Formula	Molecular Weight	Area %
6.88	1,2-Epoxy-5,9-cyclododecadiene	C_12_H_18_O	178	1.82
7.75	7,8-Dihydroneopterin	C_9_H_13_N_5_O_4_	255	1.49
10.19	2-Indanmethanol.,3Aa,4,7,7Aa-Tetrahydro-1-Hydroxy-a-Methyi	C_11_H_18_O_2_	182	2.62
10.56	3-Cyclohexen-1-nitrile, 6-methyl-	C_8_H_11_N	290	1.84
11.06	Bicyclo (7.2.0) undec-4-ene,4,11,11-trimethyl-8-methylene-, (1r- (1r*,4e,9s*)	C_15_H_24_	204	13.59
11.29	3-Ethylidene isobenzofuranone	C_10_H_8_O_2_	160	8.28
11.75	Diatrizoic acid	C_11_H_9_I_3_N_2_O_4_	614	1.58
12.32	Diatrizoic acid	C_11_H_9_I_3_N_2_O_4_	614	0.81
12.50	2-D,2-Pentadecyl-1,3-dioxepane	C_20_H_39_DO_2_	313	5.54
12.62	2-indanmethanol, 3aà,4,7,7aà-tetrahydro-1-hyd roxy-à-methyl-	C_11_H_18_O_2_	182	7.36
12.74	Diatrizoic acid	C_11_H_9_I_3_N_2_O_4_	614	2.01
13.00	(Z., Z)- α-Farnesene	C_15_H_24_	204	23.68
13.21	10,12-Octadecadiynoic acid	C_18_H_28_O_2_	276	1.98
13.69	5,7-Dodecadiyn-1,12-diol	C_12_H_18_O_2_	194	0.36
13.97	Spiro (2.2) pentane-1-carboxylic acid,2-cyclopropy1-2methy1-	C_10_H_14_O_2_	166	0.67
16.24	Acetic acid, 2,2’-[oxybis(2,1-ethanediyloxy)] bis-	C_8_H_14_O_7_	222	1.42
17.94	2,6,9,11-Dodecatetraenal, 2,6,10-Trimethyl-, (E, E,E)-	C_15_H_22_O	262	5.38
19.62	Benzoic acid,2-(1-oxopropyl)-	C_10_H_10_O_3_	178	15.78
23.36	9,12-Octadecadienoic acid (Z.Z)-	C_18_H_32_O_2_	280	1.21
24.28	Tetradecadien-4,9 ol-1	C_14_H_26_O	210	1.50
29.43	Cycloheptanol, 2-chloro-, trans-	C_7_H_13_C_1_o	148	1.08

### Experimental design and rearing conditions

All experimental methods were approved by Zagazig University’s Institutional Animal Care and Use Committee in Egypt, and all pertinent institutional policies were followed when caring for and using animals in this study (ZU-IACUC/2/F/166/2023).

One hundred and eighty *Oreochromis niloticus* (*O. niloticus*) (mean ± SE; 22.49 ± 0.06 g), all of which appeared to be in good health, were collected at a private fish farm in El-Abbassa, Sharkia, Egypt. Fish were transported alive to the Faculty of Technology and Development at Zagazig University’s Department of Animal and Poultry Production. For a 15-day acclimatization period, the fish were placed in glass aquariums (80 × 40 × 30 cm) with 60 L of dechlorinated tap water. The fish were divided into four groups, each with a triplicate (15 fish per replicate, 45 fish per group). At the start of the first week of acclimatization, the water temperature had been scheduled to 27–28 °C. During the second week of acclimation, the fish’s water temperature was gradually reduced by one degree every day until it reached 21 °C at the beginning of the feeding trial [43]. The first group (CONT) received a standard diet without any supplements. For 60 days, the second (MP10%), third (MP15%), and fourth (MP20%) groups received enriched meals containing 10, 15, and 20% of dehydrated mandarin peel, respectively. Three regular feedings throughout the day of 3% biomass each were given to the fish in each group. Over the course of the experiment, the dissolved oxygen (D.O.) concentration averaged 5.5 ± 1 mg/L, the pH level was 6.3 ± 0.1 mg/L, the nitrite concentration was 0.04 ± 0.02 mg/L, the nitrate concentration was 8 ± 0.5 mg/L, the ammonium content was 0.35 ± 0.15 mg/L, and 12 h of darkness and 12 h of light. About 25% of the water was replaced daily to maintain water quality throughout the trial period. Weighing is conducted biweekly during the study period, while samples are gathered for analysis of other parameters at the end of the 60-day feeding period.

### Growth performance evaluation

Every two weeks, the fish were weighed to assess their feed consumption, and the amount of feed given was then changed as necessary. The final body weight (FBW, g), other growth factors, and survival rate were computed [44] at the end of the experiment as follows:

Specific growth rate (SGR%) = [(Ln final weight - Ln initial weight)/60 days] × 100.

Feed conversion ratio (FCR) = feed intake (g)/weight gain (g).

Survival rate (SR) = 100 × Final number of tested fish/ Initial number of tested fish.

### Blood sample collection, biochemical and immune analysis

The fish were sedated using a 100 mg/L benzocaine solution (Al-Nasr Pharmaceutical Chemicals, Co.) [45] before sample collection. After the experiment ended (60 days), blood was drawn from the caudal peduncle region of each fish (three per replicate; nine per group) using a 25-G needle attached to a syringe without anticoagulant. The blood was left to clot for two hours at room temperature, followed by centrifugation at 3000 rpm for 15 min using a SIGMA 1–14 Centrifuge Micro Centrifuge Angle (Germany).

Biochemical analysis included the measurement of growth hormone (CAT. NO. CSB-E12121Fh, Cusabio Biotech Co., Ltd., USA), α-amylase enzyme (CAT. NO. MBS044269, MyBioSource Inc., California, USA) levels using commercial fish ELISA kits as per the manufacturer’s instructions.

For immune analysis, lysozyme activity (CAT. NO. MBS099538, MyBioSource Inc., California, USA), nitric oxide (CAT. NO. 2533, Biodiagnostic Co., Cairo, Egypt), protease activity (CAT. NO. ab111750, Abcam, Cambridge, UK), globulin (CAT. NO. MBS008318, MyBioSource Inc., California, USA), and serum levels of IL-1ß (CAT. NO. CSB-E13259Fh, Cusabio Biotech Co., Ltd., USA) were assessed using the respective ELISA kits, following the provided instruction manuals.

### Tissue collection for antioxidant status evaluation and histological indices

To collect tissue, ten fish/ group were euthanized by given an overdose (250 mg/L) of benzocaine solution [45]. The fish were starved for 24 h before euthanasia. Biodiagnostic kits from Biodiagnostic Dokki in Giza, Egypt, were used to measure the antioxidant activity. Liver tissues were immediately taken, washed with physiological saline to remove adhering blood, and kept at -80 °C until analysis. According to the manufacturer’s instructions, the homogenized liver tissue was used to assess the enzymes catalase (CAT), superoxide dismutase (SOD), and malondialdehyde (MDA).

### Histopathological investigations

For histological indices, the fish alimentary tracts were extracted, split into six recognizable segments: esophagus, stomach, anterior intestine, posterior intestine, and rectum as no clear-cut anatomical distinction were observed along the alimentary tract. Next, intestinal tissue specimens were collected from the anterior, posterior, and rectal segments (0.5-cm long for each segment per fish). Following 24 h fixation in 10% neutral-buffered formalin solution, the specimens were processed for paraffin technique where they were rinsed in distilled water, dehydrated in graded they alcohol (70% to absolute), cleared in xylene (two changes), and impregnated, and embedded in paraffin. Next, 5-µm thick tissue sections were prepared, stained with hematoxylin and eosin [46], examined by light microscope recording any histopathological changes.

### Intestinal morphometric scoring analysis

A multiparametric quantitative morphometric analysis was carried out following the protocols described by Iji et al., [47] and de Verdal et al., [48] for determining the intestinal morphological indices. Concisely, for each fish, a total of five 10× microscopic fields of round or nearly round cross intestinal sections per segment were captured. Next, these images were analyzed to determine; (1) the number of villi per image, (2) the villus height (from the base to the tip) per six villi, (3) the villus width [(villus apical width + villus basal width)/2] per six villi, (4) the villus surface area [(villus apical width + villus basal width)/2× villus height] six villi, (5), the thickness of lamina propria per image, and (6) the thickness of muscular layer per image.

### Statistical analysis

The data acquired is shown as mean ± standard error (M ± SE). Using IBM’s Statistical Package for Social Science for Windows (WINSPSS) version 25 (Chicago, USA), the significance of the difference between the tested groups was determined by ANOVA one-way, followed by Duncan’s test at *P* < 0.05. The homogeneity of variances and Shapiro-Wilk W test were used to determine whether the data were normal.

## Results

### Growth performance

**Fig. 1:**
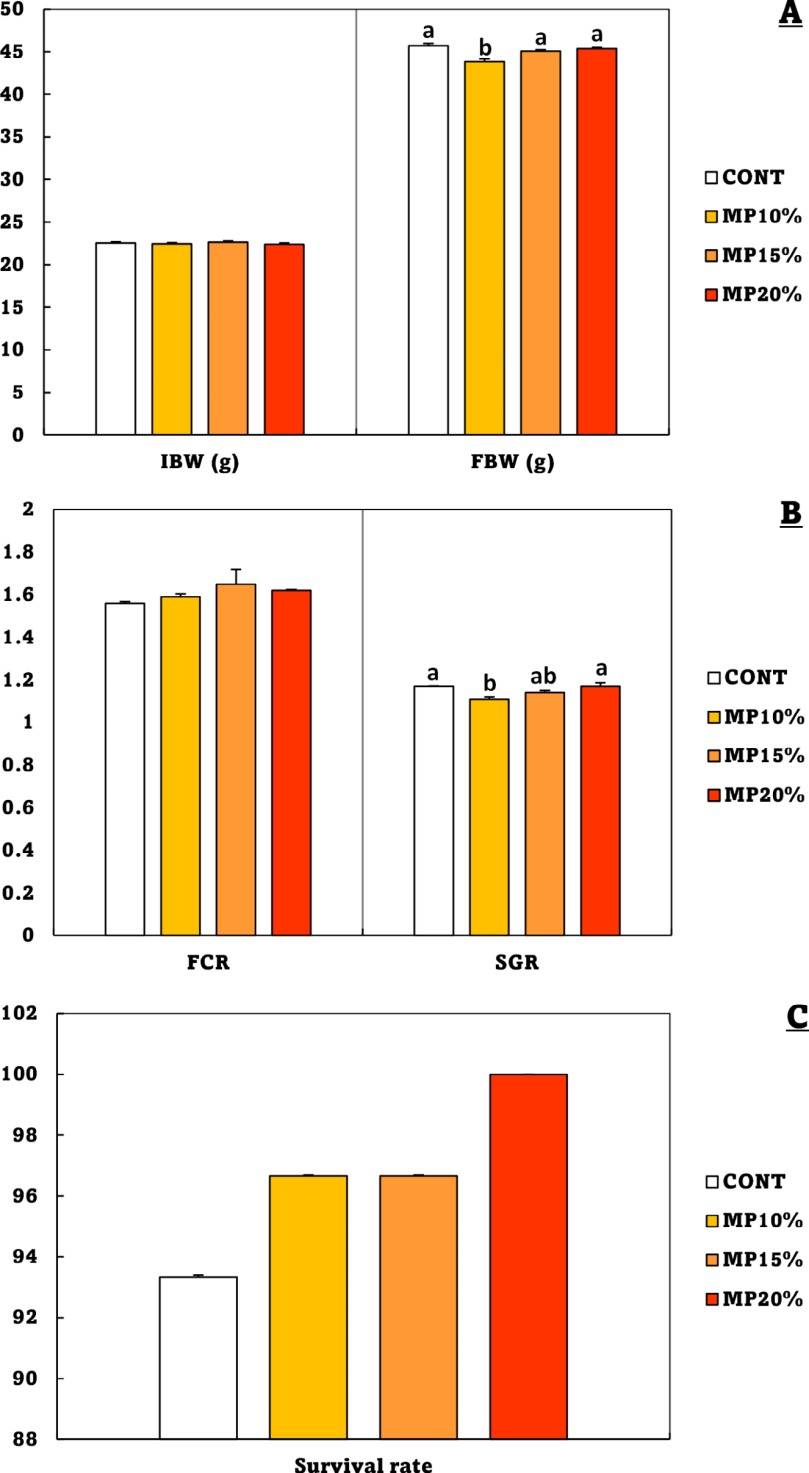
Growth performance of *Oreochromis niloticus* (mean ± SE) fed on a diet enriched with dehydrated mandarin peel for 60 days under suboptimal temperature (21 °C). **A**: Bars indicate the initial body weight (IBW), and final body weight (FBW). **B**: Bars indicate the food conversion ratio (FCR), and the specific growth rate (SGR). **C**: Bars indicate survival rate. Groups with different superscripts (a, b, c and d) are significantly different (*P* < 0.05, using a one-way ANOVA). CONT: control group, Fish fed on normal basal diet without any supplementation. MP10%, MP15%, and MP20%: Fish fed diets enriched with 10, 15 and 20% of dehydrated mandarin peel, respectively

The growth performance and survival rate of Nile tilapia fingerlings fed different levels of dietary MP are given in Fig. 1. There were no significant differences between the CONT, MP15%, and MP20% groups in the FBW and SGR, while the MP10 group had the significantly lowest final body weight. There are not significant differences between the groups and CONT group in FCR. However, the CONT group had the insignificant lowest survival rate (93.33%), while MP20% had the insignificant highest (100%), followed by MP15% and MP10% (96.66%).

### Growth hormone

**Fig. 2:**
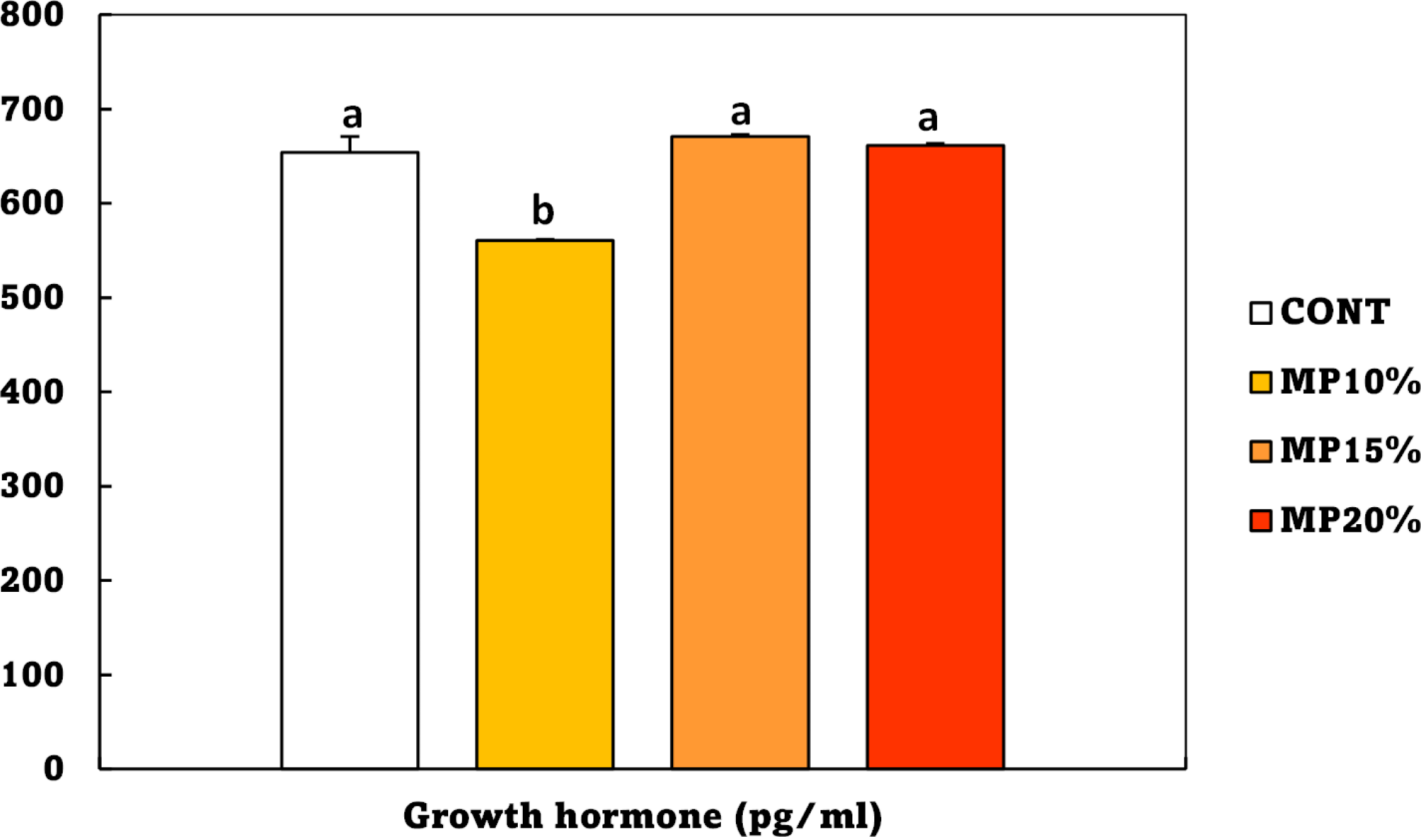
Growth hormone of *Oreochromis niloticus* (mean ± SE) fed on a diet enriched with dehydrated mandarin peel for 60 days under suboptimal temperature (21 °C). Groups with different superscripts (a, b, c and d) are significantly different (*P* < 0.05, using a one-way ANOVA). CONT: control group, Fish fed on normal basal diet without any supplementation. MP10%, MP15%, and MP20%: Fish fed diets enriched with 10, 15 and 20% of dehydrated mandarin peel, respectively

The growth hormone of Nile tilapia fingerlings fed different levels of dietary MP is shown in Fig. 2. Growth hormone increased linearly with increasing feeding MP levels. There was no significant difference in the level of growth hormone between the CONT, MP15, and MP20 groups. Fish fed a 10% MP diet had the lowest growth hormone.

### Amylase enzyme

**Fig. 3:**
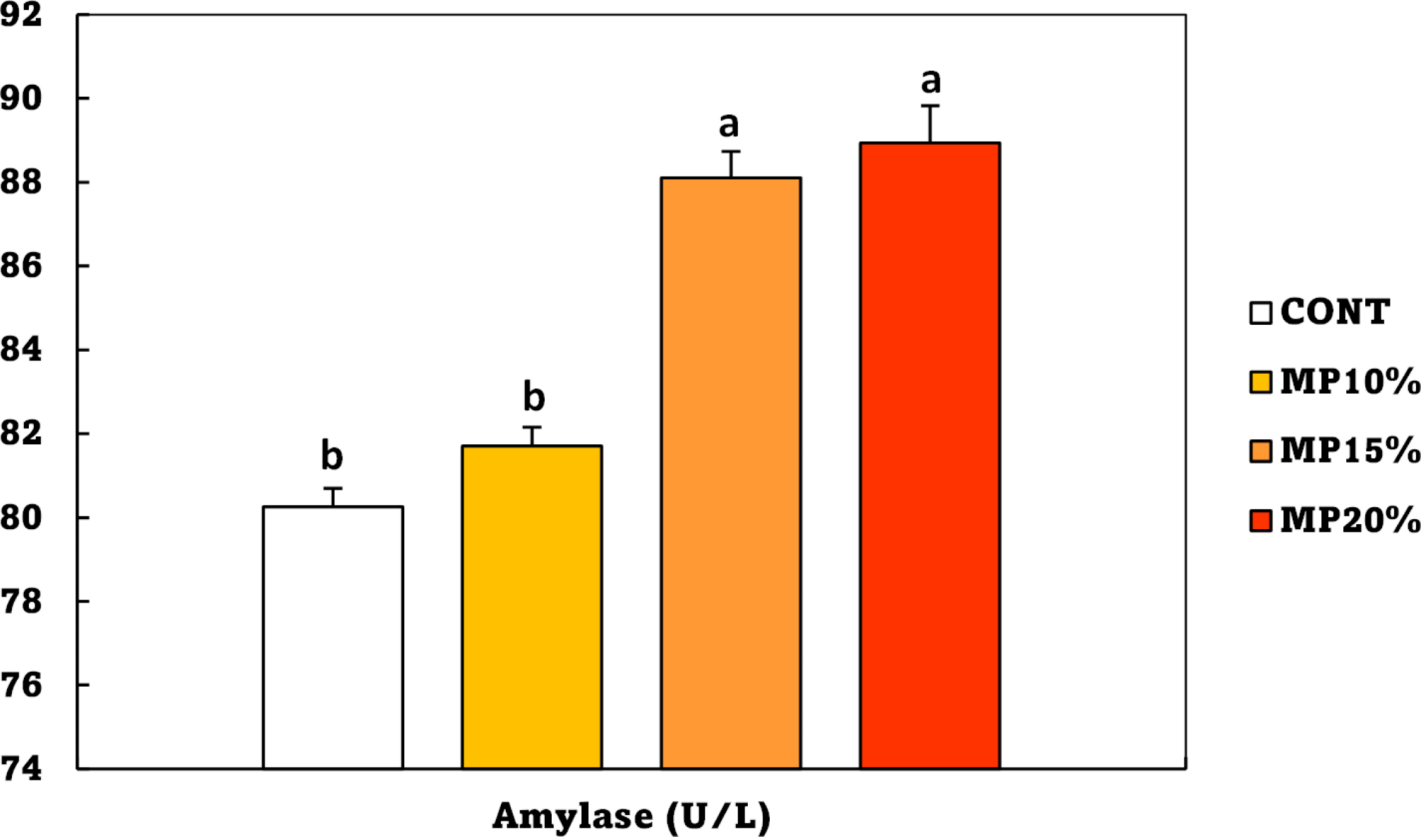
Amylase enzyme activity of *Oreochromis niloticus* (mean ± SE) fed on a diet enriched with dehydrated mandarin peel for 60 days under suboptimal temperature (21 °C). Groups with different superscripts (a, b, c and d) are significantly different (*P* < 0.05, using a one-way ANOVA). CONT: control group, Fish fed on basal diet without any supplementation. MP10%, MP15%, and MP20%: Fish fed diets enriched with 10, 15 and 20% of dehydrated mandarin peel, respectively

The amylase enzyme of Nile tilapia fingerlings fed with different levels of dietary MP is shown in Fig. 3. Fish fed diets containing 15 and 20% MP exhibited the highest level of amylase enzyme (*p* < 0.05), whereas fish fed a control diet, and 10% MP produced the lowest amylase enzyme.

### Antioxidant status

**Fig. 4:**
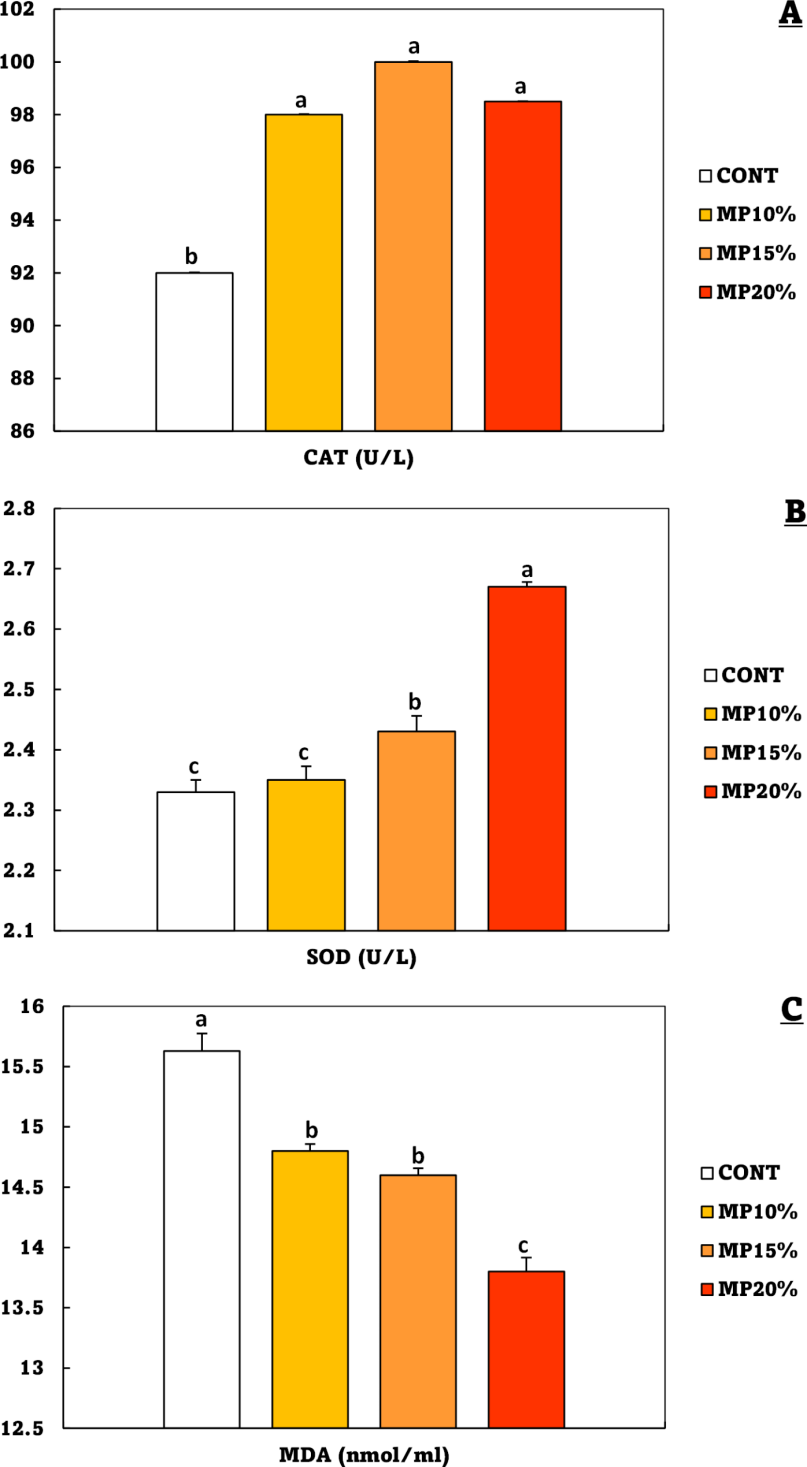
**A**: catalase (CAT), **B**: superoxide dismutase (SOD), and **C**: malondialdehyde (MDA) levels of *Oreochromis niloticus* (mean ± SE) fed on a diet enriched with dehydrated mandarin peel for 60 days under suboptimal temperature (21 °C). Groups with different superscripts (a, b, and c) are significantly different (*P* < 0.05, using a one-way ANOVA). CONT: control group, Fish fed on normal basal diet without any supplementation. MP10%, MP15%, and MP20%: Fish fed diets enriched with 10, 15 and 20% of dehydrated mandarin peel, respectively

Catalase (CAT), superoxide dismutase (SOD), and malondialdehyde (MDA) in Nile tilapia were significantly (*p* < 0.05) affected by dietary MP. The CAT activity was significantly higher than the CONT group in all groups fed on MP-supplemented diet, without a significant difference between the MP10%, MP15%, and MP20% groups (Fig. 4A). Compared to the control diet, SOD values were significantly (*p* < 0.05) higher in the MP20% group, followed by the MP15%, and then the MP10% group (Fig. 4B). On the other hand, compared to the control group, the MDA value significantly declined in the MP20% group, followed by the MP15% and MP10% groups (Fig. 4C).

### Immune parameters and interleukin-1 beta (IL-1β)

**Fig. 5:**
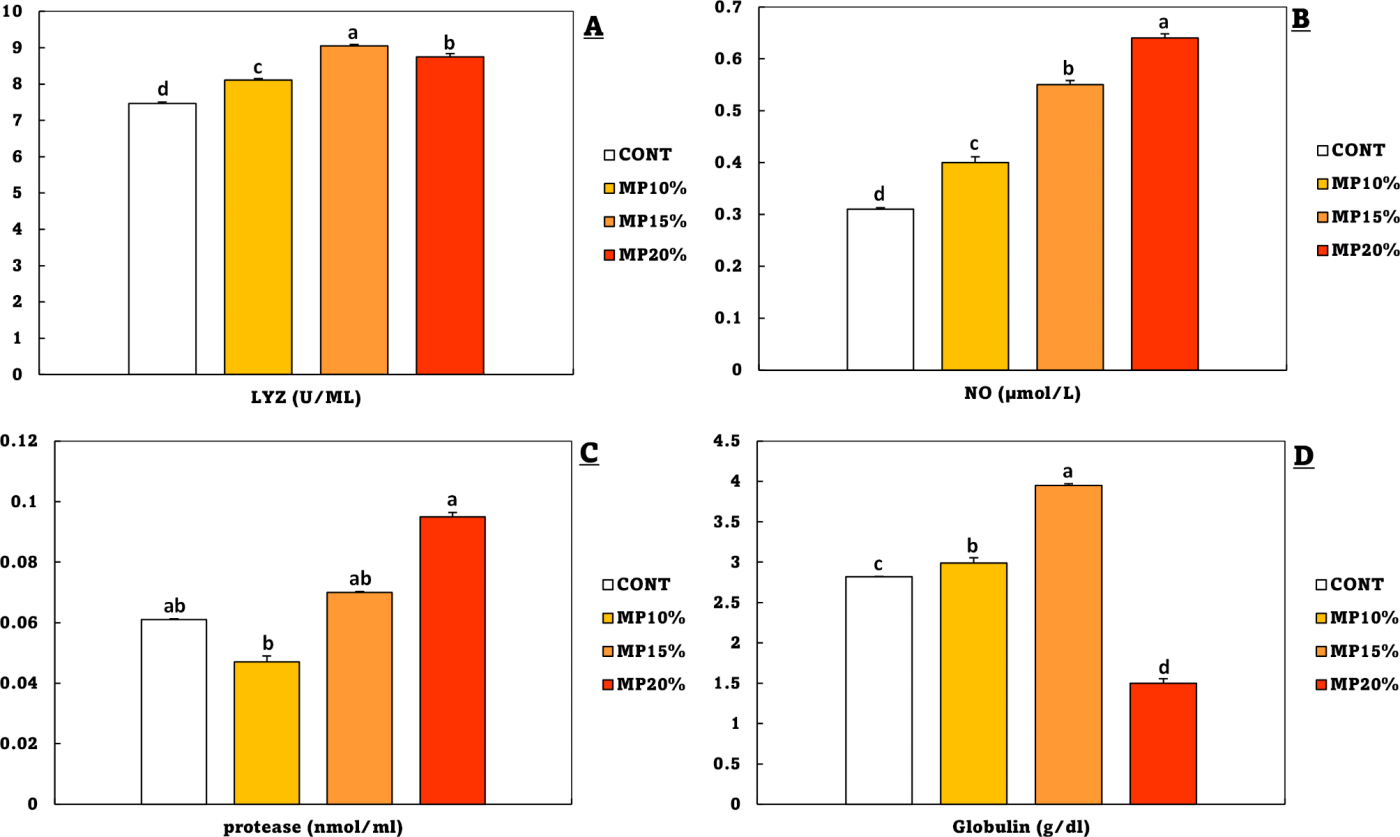
Serum **A**: lysozyme activity (LYZ), **B**: nitric oxide (NO), **C**: protease activity, and **D**: globulin level of *Oreochromis niloticus* (mean ± SE) fed on a diet enriched with dehydrated mandarin peel for 60 days under suboptimal temperature (21 °C). Groups with different superscripts (a, b, c, and d) are significantly different (*P* < 0.05, using a one-way ANOVA). CONT: control group, Fish fed on normal basal diet without any supplementation. MP10%, MP15%, and MP20%: Fish fed diets enriched with 10, 15 and 20% of dehydrated mandarin peel, respectively

The results of immune parameters were displayed in Fig. 5, which were improved in all groups that were fed MP-supplemented diet in comparison to the CONT group. The lysozyme activity was significantly higher in MP15%, followed by MP20% and MP10% (Fig. 5A). Nitric oxide activity exhibited significantly greater differences according to the pattern MP20%> MP15%> MP10% (Fig. 5B). About the protease activity, a significant increase was only recorded at MP20% compared to CONT, while there was no significant difference between MP10% and MP15% in the CONT group (Fig. 5C). The globulin levels were recorded at the highest level in MP15%, followed by MP10%, while the lowest level was recorded in MP20% compared to the CONT group (Fig. 5D).

**Fig. 6:**
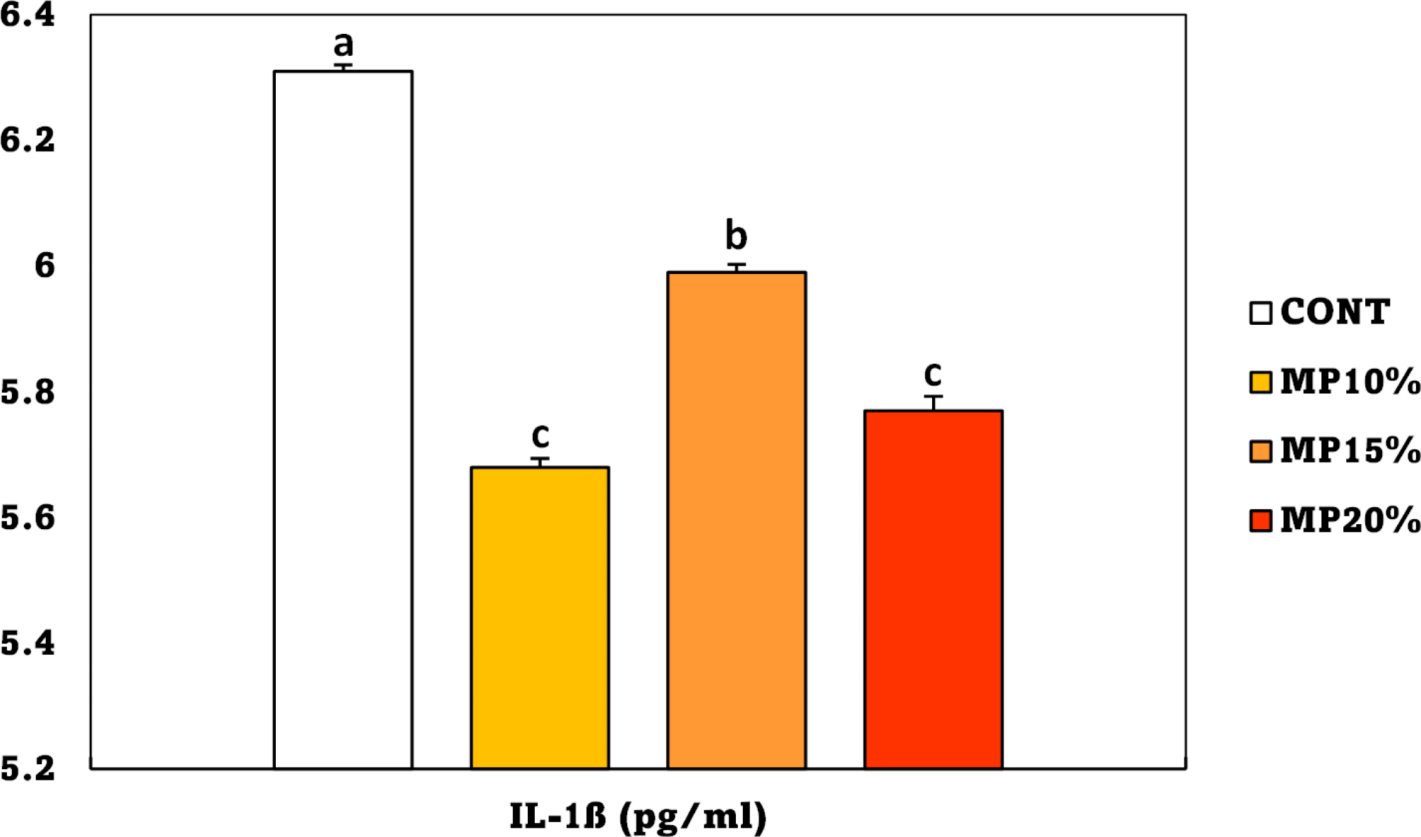
Interleukin-1 beta (IL-1β) level of *Oreochromis niloticus* (mean ± SE) fed on a diet enriched with dehydrated mandarin peel for 60 days under suboptimal temperature (21 °C). Groups with different superscripts (a, b, c, and d) are significantly different (*P* < 0.05, using a one-way ANOVA). CONT: control group, Fish fed on normal basal diet without any supplementation. MP10%, MP15%, and MP20%: Fish fed diets enriched with 10, 15 and 20% of dehydrated mandarin peel, respectively

Data in Fig. 6 showed a significant decrease in the levels of IL-1β in all groups fed a diet supplemented with MP in comparison to the CONT group. The lowest levels of IL-1β were recorded in the MP10% and 20% MP groups, followed by MP15%.

### Intestinal histology

**Fig. 7:**
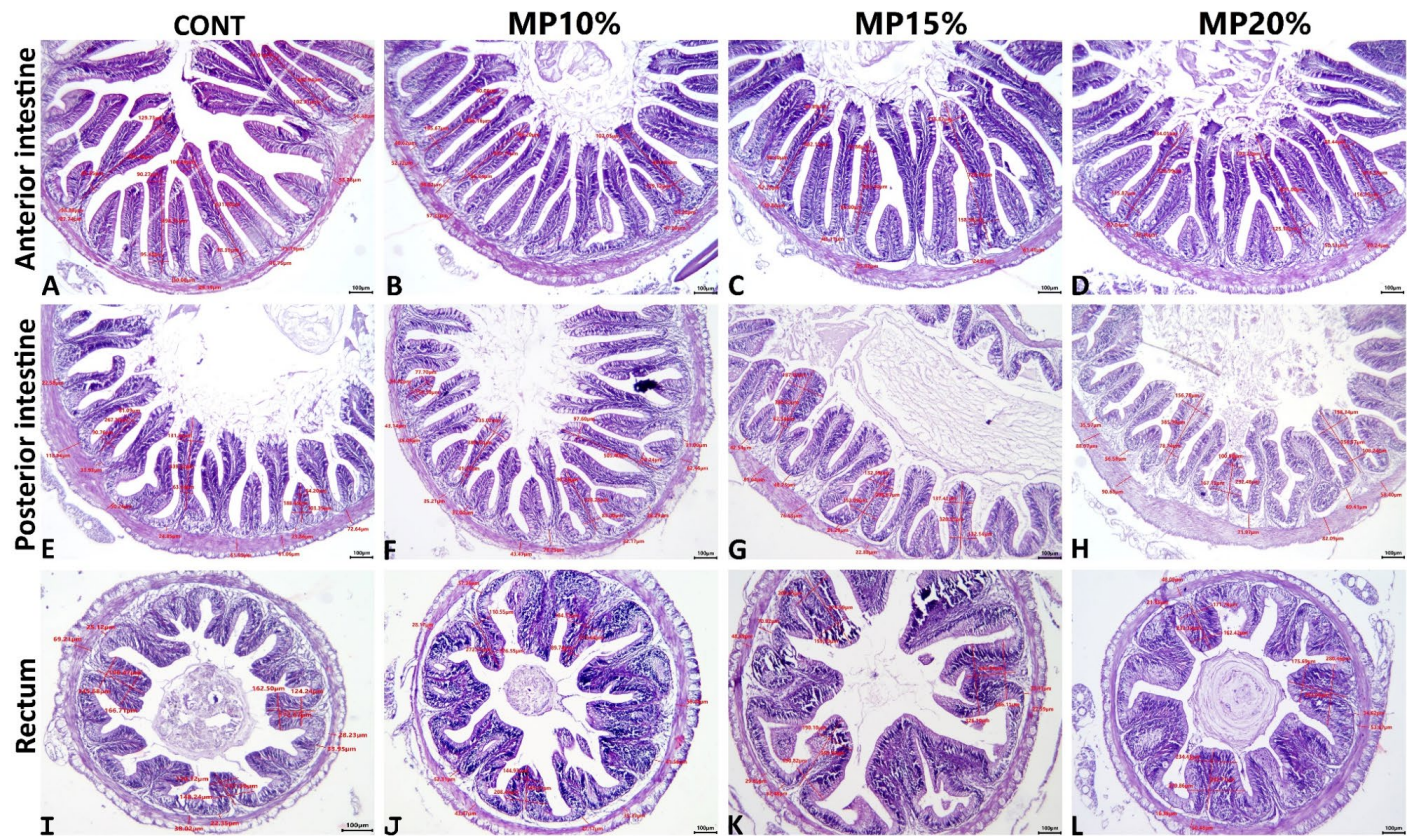
Representative photomicrograph of H&E-stained intestinal tissue sections showing the effect of dehydrated mandarin peel supplementation on the fish diet on the intestinal histology that represented by (1) no effect on the number of villi per 10× image, villus height, villus surface area, and the thickness of lamina propria, and muscular layer in the three intestinal regions among all MP-supplemented groups compared to the control group, (2) no effect on the villus width in the recti of all MP-supplemented groups compared to the control group, and (3) significant increase in the villus width in the anterior, and posterior regions in the MP-supplemented groups compared to the control group, with no significant difference to the ratio of MP in the fish diet

The image analysis declared that MP supplementation resulted in (1) no effect on the number of villi per 10× image, villus height, villus surface area, and the thickness of lamina propria, and muscular layer in the three intestinal regions among all MP-supplemented groups compared to the control group, (2) no effect on the villus width in the recti of all MP-supplemented groups compared to the control group, and (3) significant increase in the villus width in the anterior, and posterior regions in the MP-supplemented groups compared to the control group, with no significant difference to the ratio of MP in the fish diet. Subjectively, an obvious increase in the numbers of goblet cells, and numbers of mucosal-associated lymphocyte were seen particularly in the anterior, and posterior intestinal segments of all MP-supplemented groups compared to the control group, with no differences between the MP-supplemented groups. The effects of the MP supplementation on the histology of anterior, posterior, and rectal intestinal regions in all groups were shown in Fig. 7 (A-D), (E-H), and (I-L) respectively, and a detailed quantitative morphometric analysis for the intestinal histology was summarized in the Table 3.

**Table 3:** Intestinal morphometric indices of *Oreochromis niloticus* fed on a diet enriched with dehydrated mandarin peel for 60 days under suboptimal temperature (21 °C)

Intestinal segment	Criteria	CONT	MP10%	MP15%	MP20%	*P*-value
Anterior intestine	Number of villi per 10× image	12.0 ± 0.47	11.8 ± 0.41	11.8 ± 0.41	11.8 ± 0.41	0.983
	Villus height (µm)	521.7 ± 33.75	528.4 ± 32.57	530.0 ± 42.78	529.90 ± 32.80	0.998
	Villus width (µm)	96.4 ± 3.80^b^	109.1 ± 4.19^a^	109.2 ± 4.86 ^a^	108.9 ± 3.41 ^a^	0.086
	Villus surface area (µm^2^)	50418.7 ± 4085.6	577709.7 ± 4272.02	58795.2 ± 6628.25	57812.6 ± 3937.44	0.596
	The thickness of lamina propria (µm)	30.2 ± 3.94	32.7 ± 7.50	34.9 ± 2.99	33.30 ± 2.91	0.916
	The thickness of the muscular layer per image (µm)	41.8 ± 5.14	52.6 ± 2.86	52.7 ± 4.67	53.3 ± 3.15	0.156
Posterior intestine	Number of villi per 10× image	12.5 ± 0.40	12.3 ± 0.42	12.2 ± 0.29	12.4 ± 0.37	0.949
	Villus height (µm)	250.4 ± 23.01	251.2 ± 20.22	251.5 ± 23.72	252.1 ± 21.76	1.000
	Villus width (µm)	91.30 ± 4.49^b^	113.2 ± 8.32 ^a^	115.1 ± 5.69 ^a^	114.7 ± 5.16 ^a^	0.023
	Villus surface area (µm^2^)	22940.1 ± 2514.04	28,008 ± 2269.57	29694.2 ± 3723.07	29121.5 ± 31.07.81	0.368
	The thickness of lamina propria (µm)	41.10 ± 4.59	51.2 ± 5.58	52.4 ± 5.36	52.5 ± 4.33	0.320
	The thickness of the muscular layer per image (µm)	68.0 ± 6.43	71.4 ± 10.21	71.4 ± 6.08	71.3 ± 4.55	0.982
Rectum	Number of villi per 10× image	6.9 ± 0.43	6.8 ± 0.38	6.7 ± 0.36	6.7 ± 0.36	0.980
	Villus height (µm)	228.8 ± 19	239.8 ± 8.70	241.3 ± 20.85	241.2 ± 9.22	0.928
	Villus width (µm)	152.2 ± 6.78	168.0 ± 13.09	169.3 ± 6.83	169.8 ± 13.04	0.580
	Villus surface area (µm^2^)	35857.7 ± 4011.45	40150.6 ± 3423.45	41569.1 ± 4244.43	41686.6 ± 4753.68	0.729
	The thickness of lamina propria (µm)	60.9 ± 8.84	64.3 ± 4.61	66.2 ± 7.35	66.4 ± 9.28	0.955
	The thickness of the muscular layer per image (µm)	66.8 ± 6.04	68.7 ± 8.17	69.6 ± 8.14	69.5 ± 4.91	0.991

Values are represented as the mean ± SE. The means within the same row carrying different superscripts are significant at *p* < 0.05

CONT: control group, Fish fed on normal basal diet without any supplementation. MP10%, MP15%, and MP20%: Fish fed diets enriched with 10, 15 and 20% of dehydrated mandarin peel, respectively

## Discussion

Aquafeeds made from agricultural and fruit peel wastes have been the subject of many recent studies as inexpensive and sustainable ingredients [49, 50], but few have investigated how these additives affect fish raised at temperatures below ideal levels. Therefore, the purpose of this study is to evaluate the impact of adding powdered mandarin peel to the meals of *O. niloticus* grown at sub-optimal temperatures on their growth and health status.

Measuring bioactive components in fruit peels is critical for evaluating their phytonutrient, antioxidant, and vitamin content, which explains how they contribute to improved immune function, stress resilience, and growth performance [51, 52]. In the current study, (Z., Z)- α-farnesene (peak area of 23.68%) was the highest component of the mandarin peel extract. α-farnesene belongs to the class of sesquiterpenes, which is a chemical component within the terpene class [53, 54]. Farnesene has the capacity to suppress neutrophil chemotaxis and may also have immunomodulatory and anti-inflammatory modulatory properties [55]. Additionally, farnesene possesses antifungal [56], antibacterial [57], anticarcinogenic [58], antioxidant [59, 60], and neuroprotective properties [61]. The second major component in MPE of the present study, is benzoic acid,2-(1-oxopropyl)-, which is one of phenolic acid characterized by antioxidant activity [62, 63]. The next significant element in this study, Bicyclo (7.2.0) undec-4-ene,4,11,11-trimethyl-8-methylene-, (1r- (1r*,4e,9s*), has metabolic, antibacterial, bacteriostatic activities [64–67]. 3-Ethylidene isobenzofuranone is another bioactive component found in the GC-MS analysis of MPE in this study. Phthalide, another name for isobenzofuranone, is a bioactive substance that has several qualities include anti-inflammatory, antibacterial, antiplatelet, antiarrhythmic, and anticancer effects [68–71]. A review conducted by Bureš et al., [29] reported that mandarin contains a wide range of chemicals and metabolites, ranging from hundreds to thousands. Very small amounts of these substances can interfere with accurate detection. The Elmaci and Onoğur [72] study used headspace/GC/MS to assess the aroma effect chemicals in three important Turkish mandarin peel varieties: Satsuma, Bodrum, and Clementine. Limonene, terpinene, α-myrcene, pinene, and octanal were among the principal volatiles found. The chemical composition of fruit peels from several cultivars, such as Clementine mandarin, Satsume mandarin, Navel orange, and Common orange, is examined in the Bermejo et al., [73] study in Spain. The findings indicate comparable patterns for the other components, with the Satsume group exhibiting the highest concentrations of carotenoid, β-cryptoxanthin, and flavanone glycosides. GC-MS analysis of mandarin peels can identify bioactive component differences caused by a variety of factors. These include cultivar variances, regional and environmental conditions, harvesting and post-harvest treatment, extraction procedures, and genetic factors [74, 75].

Metrics measuring growth performance, such as final weight, feed conversion ratio (FCR), and specific growth rate (SGR), provide important information on how well the fish are growing overall and how effective the diet is [76–78]. Growth hormone is also essential for controlling fish metabolism, growth, and general health. Through the monitoring of growth hormone levels, we can evaluate the efficacy of diets and the ecological circumstances of fish [79–81]. According to the results of the current investigation, there was no discernible difference between the control group and the fish that were fed diets containing 15% and 20% MP in terms of FBW, SGR, and growth hormone. That is, fish diets can have a lower economic value while still sustaining growth rates. These results corroborate those of other research that assessed the impact of dehydrated lemon peels on *Clarias gariepinus* and *O. niloticus* growth performance characteristics [30] and the influence of orange peel on *O. niloticus* [82], both of which did not demonstrate statistically significant different outcomes from control groups. Conversely, some studies have shown a notable improvement in the growth performance of several species of fish that were fed on different citrus waste products overall. Dietary addition of 1% citrus bergamia peel enhanced the development, feed consumption, and overall health of *Dicentrarchus labrax* [31]. The growth performance and the regulation of growth hormone and insulin-like growth factor I genes in the muscle of *Cyprinus carpio* were found to be enhanced by 0.25% of bitter orange (*Citrus aurantium*) essential oil [32]. The dietary use of 2% lemon peel resulted in significant improvements in *O. niloticus* growth, feed consumption, and total biomass [83]. When given diets containing citrus limon peel extract, *Labeo victorianus* displayed noticeably greater weight, weight gain, and SGR, with levels increasing up to 5% [84]. These studies attributed the enhancement of growth performance to various causes such as: (i) phytochemicals and aromatic tastes, which improve diet palatability and feed intake [85]. (ii) Nutritional digestion and absorption may be improved in fish that were fed a diet supplemented with citrus peels due to their high levels of phytochemical components [86, 87]. (iii) The antibacterial components in citrus peels may also improve the health of the gut microbiota and reduce harmful microorganisms, thereby enhancing the digestive system [88]. The variation in research findings on the impact of different citrus peels on fish growth performance can be attributed to multiple factors, such as the type of citrus peel, the concentration employed, and the rearing conditions of the fish. In this work, we propose that the lack of a significant impact of MP on the growth performance of *O. niloticus* can be related to the fish being reared at suboptimal temperatures during the trial, which could potentially alter energy utilization and metabolic requirements [9, 89]. Therefore, although MP may have positive effects on health, these effects may not lead to noticeable changes in growth when the temperature conditions are not ideal.

Amylase is a crucial enzyme in the process of carbohydrate metabolism as it breaks down complex starches into simple sugars. These sugars are vital to produce energy and growth [90, 91]. In the current study, fish fed diets with 15% and 20% MP had the highest levels of amylase enzymes; fish fed a control diet with 10% MP had the lowest levels of enzymes. The study conducted by Shabana et al., [92] found that include 2–6 g/kg of methanolic extract from *Citrus sinensis* fruit peel in the diet had a substantial positive effect on the digestive enzyme activities (protease, amylase, and lipase) of *Catla catla*. Amylase levels in *O. niloticus* and mullet fish considerably increased in comparison to the control groups when they were fed diets containing 0.5% and 2% of lemon peel, respectively [83]. The fortification of *O. niloticus* diet with a 20-ml/kg phytogenic mixture extracted from *Citrus limon*, *Allium cepa*, and *Allium sativum* increased the activity of amylase and lipase [93]. This enhancement may be attributed to the phytonutrient in MP, which positively modulates gastric acidity, thereby increasing the secretion of digestive enzymes such as amylase, lipase, and proteases [93–95].

The measurement of antioxidant levels is an important biomarker for determining fish health. Superoxide dismutase (SOD) and catalase (CAT) are vital enzymes that counteract reactive oxygen species (ROS) to shield cells from oxidative damage [96, 97]. Increased concentrations of these enzymes usually signify an oxidative stress reaction, implying that the fish are facing physiological or environmental difficulties. On the other hand, MDA is a consequence of lipid peroxidation, and elevated levels of this compound indicate oxidative stress-induced damage to cellular membranes [98, 99]. In the present study, CAT activity was higher in all MP-supplemented diet groups, with higher SOD values in the MP20% group, followed by the MP15% and MP10% groups. The MDA value significantly decreased in the MP20% group, followed by the MP15% and MP10% groups. These results are similar to those of Salem et al., [88] for *Sparus aurata* that was fed an orange peel supplement; Abdel Rahman et al., [30] for *C. gariepinus* and *O. niloticus* that were fed dehydrated lemon peel powder; Lopes et al., [37] for *Rhamdia quelen* that was fed *citrus x aurantium* essential oil; García Beltrán et al., [39] for *S. aurata* that was fed dehydrated lemon peel; Laein et al., [38] for *C. carpio* that was fed Citrus lemon; Mohamed et al., [100] for *O. niloticus* that was fed bitter lemon; Harikrishnan et al., [36] for *Labeo rohita* that was fed dried lemon peel. In this study, the antioxidant status of fish may have been enhanced by the bioactive chemicals found in the mandarin peel, including farnesene, benzoic acid, and isobenzofuranone. These chemicals enhanced the production of antioxidant enzymes and eliminated free radicals [29, 101].

The levels of nitric oxide in fish indicate the inflammatory state and amount of immunological activity [102]. Lysozyme, an enzyme possessing bacteriolytic capabilities, serves as an indicator of innate immunological capacity [103]. Antiprotease activity refers to the ability to suppress proteolytic enzymes, which is essential for preserving tissue integrity during immunological responses [104]. Globulin levels, which are made up of several immunological proteins, provide information on the state of the immune system as a whole as well as the capacity to create antibodies [105]. In the current study, the immunological parameters (lysozyme, nitric oxide, and antiprotease activities) are significantly higher in the MP20% and MP15% groups in comparison to the control group. The lysozyme activity, phagocytic activity, and total leucocytes of *O. niloticus* significantly increased when fed on dehydrated lemon peel powder (1–2%) and bitter lemon peels (0.75–1%) [30, 100]. *Labeo rohita* lysozyme activity, respiratory burst activity, phagocytic activity, peroxidase activity, alternative complement pathway, and serum immunoglobulin M (IgM) increased significantly when fed a diet supplemented with dried lemon peel at 5 g/kg [36]. Sadeghi et al., [106] found that *Cyprinus carpio* that were fed a diet with lemon peel (1.5–3%) had a significant increase in serum lysozyme activity. The inclusion of 1.5% dried lemon peel (DLPP) in the diet of *Oncorhynchus mykiss* resulted in a significant increase in total immunoglobulin, lysozyme activity, and alternative complement (ACH50). Additionally, fish fed with 0.5% DLPP demonstrated an increase in immunoglobulin M levels [34]. The presence of phytonutrient components, particularly farnesene, which have immunomodulatory, anti-inflammatory, and antioxidant properties, may be responsible for this immune enhancement [55, 61].

IL-1β, a pro-inflammatory cytokine, is an important indicator for fish health [107, 108]. In this study found a significant decrease in IL-1β levels in all groups fed a diet supplemented with MP, especially at 10 and 20% compared to the CONT group. The reduction in IL-1β levels suggests that the diet supplemented with MP may possess anti-inflammatory properties and might enhance the fish’s general health while reducing suboptimal temperature stress. Gosslau et al., [109] proved that orange peel extracts enriched with bioactive polymethoxy flavones significantly reduced the expression of inflammatory genes such as COX-2, TNF-α, ICAM-1, NFκB, IL-1β, IL-6, and IL-8. According to a study conducted by Hamdan et al., [27], the waste from Egyptian Murcott mandarin cultivars, particularly at the leaves, has anti-inflammatory properties against the enzymes cyclooxygenases (COXs) and 5-lipoxygenase (5-LOX). This is because the waste contains flavonoids, phenolic acid derivatives, free organic acids, flavonoid aglycones, and flavonoid glycosides. Furthermore, research of Pallavi et al., [110] shows strong anti-inflammatory and anti-nociceptive action of citrus fruits including Lime, Orange, Sour Orange, Pomello, and Citron. The phytoconstituents facilitate this powerful activity, rendering them efficacious therapeutic agents for acute inflammations. *Sparus aurata* that were served a diet containing 3% dehydrated lemon peel for a period of 15 days exhibited a significant increase in the expression of the IL-1β gene. Nevertheless, this up-regulation was not observed after 30 days, and the expression of the genes was comparable to that of the control fish [39]. On contrary, the expression of IL-1β was markedly increased in both unchallenged and *Aeromonas sobria* challenged *Labeo rohita* fish that were fed diets containing 2.5 and 5 g/kg of dried lemon peel for 60 days [36]. The variation in IL-1β levels may differ between the different studies depending on the type of citrus peels used, fish species, dosage, trial duration, and experimental settings.

Histology is an important method for studying the impact of feed additives on fish intestinal morphology, as it provides information about cellular and tissue changes. This helps us understand how additives affect nutritional absorption, intestinal health, and overall fish growth [111, 112]. The present study indicated that MP supplementation had no significant effect on the number of villi, villus height, surface area, thickness of the lamina propria, or muscle layer in the three intestinal regions as compared to the control group. However, it did widen the villus in the anterior and posterior regions. Subjectively, all MP-supplemented groups had more goblet cells and mucosal-associated lymphocytes in the anterior and posterior intestinal segments than the control group, with no difference across the MP-supplemented groups. Increased width of intestinal villi as well as goblet cells and mucosal-associated lymphocytes enhances the efficiency of digestion and absorption in the gastrointestinal tract, facilitating the uptake of essential nutrients such as amino acids, fatty acids, and glucose [100, 113]. This structural adaptability also improves enzymatic function and intestinal health, since research indicates that changes in the shape of the villi enhance the absorption of nutrients and promote better growth performance [114, 115]. *O. niloticus* intestinal morphometry, such as villi length, inter-villi spacing, and goblet cell counts, was considerably enhanced by dietary supplements including essential oil extracts from sweet orange (OEO) and/or lemon (LEO) peels [100]. The height and density of *O. niloticus* intestinal villi increased as the amount of orange peel (OP) in the diet increased, with the best results observed at a concentration of 2 g OP/kg. However, at a concentration of 4 g OP/kg diet, the height and density of the intestinal villi continued to increase until it significantly impacted food mobility [116]. The intestine of Caspian white fish (*Rutilus frisii kutum*) fed with 25 mg/kg of grapefruit peel extract (GPE) had the greatest levels of villus width, height, and surface area [117]. Dietary supplementation with *Citrus x aurantium* essential oil (EOCA), primarily at a level of 0.5 mL EOCA/kg diet, positively impacted the intestinal morphology of *Rhamdia quelen* [118].

## Conclusion

The study concluded that introducing 20% MP into tilapia’s diet is a successful technique for improving their health at temperatures below 21 °C. Despite no significant differences in final body weight or specific growth rate between the groups, tilapia fed 15% and 20% MP had higher amylase enzyme levels, as well as antioxidant, lysozyme, nitric oxide, and protease activity. These fish also showed lower levels of the pro-inflammatory cytokine interleukin-1 beta, broader intestinal villi. As a result, a 20% MP diet improves tilapia’s health and resistance in suboptimal conditions.

## Data Availability

All the data generated or analyzed during this study are included in this published article.
